# From *Necrocene* to *Naíocene*—promising pathways toward sustainable agri-food systems

**DOI:** 10.1007/s11625-022-01255-3

**Published:** 2022-11-15

**Authors:** Markus Keck, Andrew Flachs

**Affiliations:** 1 Center for Climate Resilience, Augsburg University, Universitätsstraße 2, 86159 Augsburg, Germany; 2 College of Liberal Arts, Purdue University, 100 North University Street, West Lafayette, IN 47907 USA

## Introduction

By September 2022, deadly overflooding of the Indus River in Pakistan left at least 1200 people dead and nearly a third of the country under water. This is just another impression of what climate change actually means for the habitability of this planet for human societies. According to the Pakistan Meteorological Department, the 2022 monsoon was the wettest since record keeping began in Pakistan in 1961. More than two million acres of agricultural land were submerged under water, which wiped out crops, razed buildings, and engulfed entire villages. Months into the Russian army’s invasion of Ukraine, key food, oil, and fertilizer infrastructure disruptions have led to sharp increases in food prices and global inflationary pressure that hit hardest communities where food is a larger percentage of household budgets. Massive late summer droughts in France and Germany, the drying up of the Po River in Italy, and historic heat waves across the US from Phoenix to North Platte to San Antonio to Chicago have exposed with vigor the challenges that extreme weather events and political mismanagement pose on agriculture and the production of our most basic resource: food. Whichever region of the world we look at, one thing is clear from this combination of sociopolitical disruption and ecological change: we have a problem.

To better comprehend this problem, environmental scientists have come up with the notion of the “Anthropocene,” denoting the most recent geological epoch as a period during which human activity has become the dominant influence on climate and the environment (Crutzen and Stoermer 2000; Lewis and Maslin 2015). According to the scientists, the Earth system has remained stable throughout the Holocene, despite some natural environmental fluctuations over the past 11,700 years (e.g., rainfall patterns, vegetation distribution, nitrogen cycling). During the Holocene, key biogeochemical and atmospheric parameters of the Earth system have fluctuated within a relatively narrow range (Rockström et al. 2009). However, since the industrial revolution, those indications have changed tremendously. Humans have pushed the planet outside the Holocene range of variability for many key Earth system processes, which has increased the risk of catastrophic environmental change at a global scale—especially from the 1950s onwards—and which might cause even the collapse of present-day global civilization (Steffen et al. 2015a, b).

Following Johan Rockström, scholars argue for the concept of “planetary boundaries” to provide decision-makers a guideline for steering the Earth system back into the Holocene stability domain (Rockström et al. 2009). These boundaries are human-determined values of certain control variables that relate to the most vital Earth system processes (i.e., climate change, change in biosphere integrity, stratospheric ozone depletion, ocean acidification, phosphorus and nitrogen flows, land system change, freshwater use, atmospheric aerosol loading, and introduction of novel entities) and together define a safe operating space for human activity to proceed without risking the non-negotiable preconditions for human life on Earth to be jeopardized (Steffen et al. 2015b).

Clearly, the present way of how food is cultivated, manufactured, transported, and consumed is one of the major factors behind the ongoing push of the planet outside the Holocene range of variability (Weis 2010). Campbell et al. (2017) have found that the agri-food system is the major driver of two planetary boundaries currently at high risk: biosphere integrity and biogeochemical flows of phosphorus and nitrogen. Furthermore, the agri-food system is responsible for further two boundaries at increasing risk (i.e., land system change and freshwater use), and needs to be addressed as a significant contributor to a third one (i.e., climate change). As their study confirms, nothing less than a radical transformation of the entire agri-food system is required since all involved activities, from agriculture, through processing, logistics, and retail, to consumption, affect planetary boundaries to some extent, and thereby offer a wide range of mitigation possibilities (Ingram 2011; Ingram and Porter 2015).

At first glance, the issues at stake seem to be obvious. Human societies, through their agri-food systems, act upon climate and the environment in ways that are clearly unbearable. Against this background, solutions must be found to reduce greenhouse gas emissions, increase the efficiency of resource use, and protect agro-biodiversity. Yet, at second glance, follow-up questions pop up: What type of solutions are needed? What variation exists within our planetary *Anthropos* to distinguish individuals and institutions who are uniquely vulnerable or responsible for planetary change? Can the challenges of the Anthropocene be met by means of technical innovations alone (Jasanoff 2015; Nightingale et al. 2020)? And how far does the economy need to be adjusted to help human civilization to return to its safe operating space (Knappe et al. 2019; Koretskaya and Feola 2020)? Given these questions, the last years have seen a lively debate about the validity and usefulness of the term “Anthropocene” itself. Critics have argued that attributing global environmental change to a universalized *Anthropos* risks ignoring the role of structural inequalities along the lines of class, race, gender, and geography in producing this change (Malm and Hornborg 2014), proposing instead that our era’s profound socioecological shifts are functions of capital (Moore 2017), plantations (Wolford 2021), Cthulhu (Haraway 2016), and, in the wake of the global COVID-19 pandemic, viruses (Fernando 2020). Furthermore, singling out a species as irreversibly dominant might inhibit political action by naturalizing political–ecological crises, normalizing narratives of control as progress, institutionalizing human mastery, or reifying a false division between humans and the biophysical world of which they are part (Simpson 2020; Swyngedouw and Ernstson 2018). In the light of these critiques, Reisman and Fairbairn (2021) have argued for understanding the Anthropocene not only as a set of physiological phenomena of the Earth system, but also as an existential crisis of modernity. To get a grip on what this means and what implications grow out of this observation, we need to delve deeper into the origin of the Anthropocene, which is closely related to the discussion of its root causes.

## The Anthropocene: origins and implications

Environmental scientists have presented the development path of the Anthropocene as a matter of natural history. In this way, Crutzen (2002), Steffen et al. (2011) and others have described the rise of the “geology of mankind” as a progression of technical revolutions that serve to structure a sort of unifying world history. This world history starts with the appearance of *Homo sapiens* as a species that have existed for well over 95% of its 300,000 years history as hunter–gatherers (Hublin et al. 2017). During that time, the first humans had detectable impacts on their environment (e.g., fire-stick farming and hunting of mega-fauna) that, however, registered only slightly at the global scale. In consequence, the functioning of the Earth system continued relatively unchanged (Steffen et al. 2011). About 10,000 years ago, near the onset of the Holocene, agriculture emerged first in the fertile crescent and then independently across the world. Over the next several thousand years, agriculture became linked to a more sedentary lifestyle, the development of cities, land-clearing, distinctive shifts in human-influenced ecological niches (Smith and Zeder 2013), and the creation of larger hierarchical societies. These activities measurably increased the atmospheric CO_2_ concentration, but due to the constrained availability of energy resources that increase was not enough to raise the CO_2_ concentration beyond natural variability (Steffen et al. 2007).

Another noticeable change began in the late eighteenth century, when the so-called industrial revolution characterized by mechanization and increasing use of fossil fuels spread from England to other world regions (Crutzen 2002). Extensive land-clearing and the industrial fixation of nitrogen from the atmosphere for fabricating chemical fertilizer drove food production to unprecedented heights, while newly installed sanitation and health systems increased life expectancy and facilitated population growth. At the same time, fossil fuel-based manufacturing not only led to the massive procurement of material goods, but also raised the CO_2_ concentration in the atmosphere so that, by the early twentieth century, this concentration for the first time exceeded the former Holocene variability (Steffen et al. 2011). By the middle of the twentieth century, the present-day stage of the Anthropocene (i.e., the so-called great acceleration) began, characterized by close to exponential growth rates of nearly every variable in the Earth system, including human population, the use of primary energy resources, water, or fertilizer, the concentration of CO_2_, N_2_O, and CH_4_, the rate of ocean acidification, and the rise of the Earth’s surface temperature (Steffen et al. 2015a).

Several aspects become obvious through this natural history of the Anthropocene. First, in these narratives humans are mainly addressed as biological species rather than political creatures. For this reason, authors confront their readers with rather black-boxed descriptive categories such as population size or resource consumption instead of providing thick descriptions of different cosmologies, artisanal practices, or political organization patterns throughout history (Swyngedouw and Ernstson 2018). Second, this natural-historic account emphasizes so-called technical revolutions (i.e., agricultural revolution, industrial revolution, etc.) while minimizing the philosophical and scientific roots of the materials fueling these revolutions in the first place (i.e., coevolutionary relations between humans and other species that shaped domestication, agriculture, and pastoralism; coal and steam engines in the first industrial revolution; oil, petrochemicals, and electricity in the second industrial revolution), not to mention the infrastructure necessary to make use of them (Moore 2017). Instead, resources and technologies are presented as closed totalities, always already part of some pure “nature” or “society” category (Latour 1993). Third, and related to the former point, the natural history of the Anthropocene rests upon what Moore (2017, 2018) has called the “green arithmetic:” a way of conceiving nature and society not only as fundamentally different and distinct from (even though connected to) each other, but also as being inhabited mainly by substances (e.g., human bodies, natural resources, etc.) that are fully describable and controllable by scientific, technical means. By privileging substance over relations, this arithmetic undergirds the widely spread narrative in which “humanity acts upon nature.” In such a story, society (e.g., technology, economy, etc.) and nature (e.g., ecosystems, hydrology, etc.) are abstractable from their embeddedness in socio-geo-ecological interrelations (Nightingale et al. 2019).

The green arithmetic with its distinction of nature and society is not only a statement that is questionable from an ontological point of view but must also be seen as the root cause of severe maldevelopments worldwide with devastating effects on human and non-human societies alike. Some of these pitfalls are ontological: as Moore (2017) rightly mentions, the green arithmetic has its philosophical roots in the Cartesian distinction of mind and matter (i.e., res cogitans and res extensa), a philosophy that is often said to be the beginning of modernity. Descartes (2016) derived this distinction from his famous thought experiment, in which he imagined a malicious demon who doubts everything that bodily senses can perceive about reality. By following the demon, Descartes starts doubting the existence of all things around him and lastly arrives at the point where he even doubts the existence of himself. In this time of utmost misery, he luckily spots a self-saving conclusion: as long as he thinks that reality is non-existent, he himself continues moving this thought. Based on this finding he concludes that, at least, he must exist even if no absolute certainty can be reached regarding the external objects. With this basic distinction between *res cogitans* (him thinking) and *res extensa* (objects external to his mind) Descartes drove a wedge into Western thinking that has been inscribed deeper and deeper into modern thought over centuries (Latour 1993) so that today, distinctions of society and nature, human and non-human, or mind and matter seem like natural givens in many parts of the world. Yet despite its long history, this distinction is flawed. Descartes’ “*Cogito ergo sum*” and his binaries exhibit a logical error when we return to issues of politics and relations. This bare thought would never have appeared as such without certain preconditions: language as a means of communication, philosophy as a more or less institutionalized way of thinking in French intellectual circles, patriarchal and racial norms that allowed Descartes to pursue mathematics and philosophy in colonial France and the Netherlands, the body physiology that made his thought emerge, and the web of life that let that body emerge in the first place.

Descartes’ flawed ontology would not be problematic, if it were only discussed among arcane groups of philosophers. But unfortunately, this has not been the case. This brings us to the second point of the statement above. In fact, the Cartesian distinction of society and nature was an ontological and practical precondition for the global civilization’s main principle of organization, capitalism, which came into being alongside the force and violence of colonialism. In dividing the world into a “natural” and “social,” Cartesian ontology provided a frame for colonial-capitalists to see that natural, including commodity resources but also other human beings, as both outside their social world and as a means for enrichment. In this sense, Descartes provides a scientific justification for both colonial hierarchies of life as increasingly removed from states of nature and an economic imperative to capture value from this natural, that is to say uncolonized, state. While Weber (2002) saw the origins of capitalism in protestant ethics, with discipline, frugality, and ingenuity as its main corner stones, Marx (1867) rather saw it in a history of violent expropriation, colonial expansion, and racialized enslaved labor (Burnard 2015). This history began inter alia with the sugar plantation systems on Madeira, where the first signs of the modern sugar-slave-nexus took shape, was later moved to São Tomé and northeastern Brazil, and spread from there to many other regions in the world until today (Mintz 1985). Wherever the first plantation systems were installed, mass killings and an unprecedented destruction of the environment followed (Haraway 2015). For instance, only 40 years after Madeira’s sugar boom started, over half of the island’s accessible forests had been cleared. Similarly, a third of São Tomé was deforested only 50 years after the advent of the first plantations. And, finally, from nearly 240,000 enslaved African people who arrived in northeastern Brazil in the half-century after 1600, not more than 60,000 were alive in 1650 (Moore 2017). Thus, as Moore (2017) impressively shows by means of numerous historic references like the one mentioned, the history of capitalism rests upon colossal environmental devastation and mass killings—a reason why he declares the present era to be better called “Necrocene,” the age of death and extreme extermination (McBrien 2016).

The Necrocene rests upon two principal processes, which are both related to the Cartesian society/nature dichotomy, namely the exploitation of cheap labor and the appropriation of cheap energy, food, and raw materials (Moore 2018). The exploitation of cheap labor is built upon a racial and gendered formation in which certain groups of people (e.g., indigenous people, most women, Africans) were expelled from the sphere of humanity and rather treated as part of nature. As a part of nature under a colonial extractive regime, such life was devalued in the ethical sense that these people were not given dignity or respect by their oppressors and in the economic sense that their labor, knowledge, and acts were not compensated for their larger societal worth. In that era, which started long before the industrial revolution, the said dichotomy was realized by a distinction of European civility and non-European savagery, and by a highlighting of male labor and productivity that necessarily obscured female care and reproductive labor. Such distinctions then became the building blocks of a multi-faceted process of devaluation of work performed by certain humans that evolved not only around matters of class, but also of race and gender, and helped capital to accumulate its first surpluses. The appropriation of cheap energy, food, and raw materials, interestingly, works in quite similar ways. It rests upon a segregation of nature from economic processes that leaves economies describable by figures on productivity, efficiency, and profitability, while nature is conceived as ready-at-hand resources to be mined, processed, and consumed. In this way, the society/nature dichotomy translates into a process of devaluation of the work and energy of other living organisms and entire geo-bio-chemical processes—what we call “ecosystem services” today (Millennium Ecosystem Assessment 2005)—for the sake of generating growth rates that alone serve the end to tell capital’s story of endless growth. Together, the two processes of exploitation and appropriation generate what Moore (2017) calls “cheap nature”—a process that turns the manifold living interspecies connections into dead abstractions that only serve capital to accumulate profits.

## From Necrocene to Naíocene: promising pathways toward the future

The society–nature dichotomy of green arithmetic, and the “humanity acts upon nature”—narrative that such arithmetic offers, is useful in supporting a simplistic narrative that justifies colonial hierarchies and capitalist extractions of life. Yet in practice, the interrelations of nature culture (Haraway 2003) in a world ecology, and our understandings of them, are rather products of a complex historical process that involves philosophies, technical devices, and human and nonhuman work/energy entangled in multi-faceted and ever-changing assemblages we have addressed as capitalism and colonialism. The major shortcoming of this dichotomy is that it is ontologically misleading and obscures that society and nature are not two distinct classes of reality, but rather co-constituted (and co-constituting) ingredients of the vibrant web of life found on planet Earth. Now, if we do not want to stop reflecting upon the origins of the present-day crisis, but want to examine possible other futures after the Anthropocene, how do we proceed?

In this special issue, which interrogates sustainability in contemporary agri-food systems, we ask what benefits arise from an analysis of humans as full members of the web of life experiencing structural differences in precarity and responsibility for global ecological change. Many colonial-capitalist agricultures have proven to be disastrously unstable as a result of their intrinsic political and ecological violence: newly vulnerable wildfire landscapes from California to Australia stem not only from emissions-related global climate change but also from the genocide of Indigenous caretakers; agrarian crises in the United States, Brazil, and India stem not only from water extraction but land dispossession in the interest of monocrop expansion. As Black feminist and other scholars of the capitalist plantation have shown across centuries (Davis et al. 2019; Jegathesan 2021; Li and Semedi 2021; McKittrick 2013; Wolford 2021), these systems tried to replicate hierarchies of race, gender, and class in extractive farm fields through violence and surveillance. And yet, those scholars also describe how plantations failed to completely sever links of community and care. It is thus in repairing and reorienting to these connections that the Necrocene, the era of death and destruction, falters and a new era of living and caring offers solutions to a sustainable future (Blanco-Wells 2021). We call this new era the “Naíocene.”

Naíocene is a neologism built from the old Greek ναίειν (naíein), to live, dwell, and be situated. It differs from the two other notions related to life, βῐ́ος (bíos) and ζῷον (zôon), used to indicate the science of nonhuman organisms and populations, in highlighting living as an active process built on interrelations and care with other life forms. At the same time, the Naíocene can be said to be the antipode of the Necrocene. It refrains from separating reality, but embraces the entanglement of philosophy and imaginations, technical devices and scientific practices, as well as work/energy performed by human and nonhuman people. In other words, it follows Haraway’s (2016) suggestion to staying with the trouble and it speaks out an invitation to scholars worldwide to engage in new forms of scientific storytelling that leaves the seemingly self-perpetuating machinery of capitalism and colonialism behind and offers promising pathways toward creating a planet worth living.

A key consequence of a binary green arithmetic that separates society, or capitalism, and nature is in how this Necrocene ontology identifies the problems and solutions of the global crisis. If politics can be separated from ecology, then a range of technical fixes become logical within that narrative: everything from neo-Malthusian calls for population decrease to state regulations to geoengineering (Nightingale et al. 2020). A Naíocene might instead see social and environmental processes as both producers and products of the web of life, with an assumption that continued existence within the web of life involves coevolution in a changing world. Mounting evidence shows that human systems have coevolved with the landscapes around them for thousands of years in ways that shape genetic inheritance as well as social infrastructure—not merely through domestication and agriculture (Smith and Zeder 2013; Stephens et al. 2019) but also through complex interactions with ecosystems that can lead to the flourishing or destruction of life (Barthel et al. 2013). Scientific processes that value objectivity and experimental design are only now beginning to recognize how care work can shape forests (Armstrong et al. 2021), tidal environments (Lepofsky et al. 2017), gardens (Nazarea and Gagnon 2021), desert landscapes (Nabhan et al. 1983), and seeds themselves (Deppe 2021; Mueller and Flachs 2022; Soleri et al. 2002) that in turn help to structure socioeconomic activity. Indeed, the Naíocene allows us to ask how such environments were always shaped with communities of practice.

While a green arithmetic perspective can separate social and natural elements to effectively highlight the ecological consequences of a political economy, linking them through a Naíocene approach can be even more revealing of the lives that can flourish or perish in these relationships. Sugar and cotton plantations are dramatic examples of colonial-capitalist ecologies, with consequences for industrial agriculture into the twenty-first century. Built on land stolen by broken treaty or military force, plantations effected an infrastructure of both carceral surveillance and assembly-line capitalism. Sugar and cotton economies embraced a worldview of white supremacy, underwritten by imperial governors, to devalue the labor and knowledge of African and Indigenous American people, effecting an ecology of monoculture and control for nonhuman lives across the landscape (Beckert 2014; McInnis 2019; Mintz 1985). The cheapening of human life was only possible in a larger Necrocene ontology that both separated humans from and then devalued environmental resources themselves. Importantly, plantation ecology continues to produce hierarchies of race, class, gender, labor, and biological simplification (Li and Semedi 2021; Reese and Sbicca 2022) this time subsidized by the state (Stone 2022). The Necrocene welcomes plantations and industrial monocrop ecologies as efficient tools to increase production for international trade and speculation, with the health and wellbeing of humans, our organization, and our ecologies rendered externalities or necessary sacrifices. Yet as neither the Necrocene nor capitalism were ever totalizing, scholars have shown how communities of care and multispecies thriving coexist with plantations against violent odds (Carney 2020; Heynen 2021; Jegathesan 2021).

Despite the enormous pressure of the Necrocene on the web of life, the ecology of capitalism is never as fully totalizing as the system claims to be. This failure to commodify all life is, on one hand, a source of infinite resource frontiers. Blanchette’s (2020) study of American pork, for example, shows the horrific lengths to which pork capitalism goes to find new value from pigs, workers, and land. When agri-food futures demand limitless growth, such resource frontiers must be pursued, captured, and financialized. On the other hand, the pursuit of capital growth against the other realities of farming, ranging from the replenishment of soil fertility to the desire to see children play on the farm, sets up paradoxes of stability and growth in agriculture under capitalism. Ethnographers have chronicled historical and ongoing means of resistance to colonial-capitalist ecologies in ways that suggest the Naíocene’s persistent influence: mushroom foragers processing trauma and forming connection in disturbed landscapes (Tsing 2015), cotton workers demanding recognition of their labor in post-industrial Mumbai (Finkelstein 2019), and enslaved people finding moments of reprieve through the tastes of home (Carney 2020) each find ways to restore relationships of care and love with other people *because* they perform acts of ecological reparation. Such diverse economies (Gibson-Graham 2008) coexist because capitalist relations fail to describe the porous connections of human and natural systems. If, as Moore (2017) suggests, our Necrocene agri-food systems stem from an ontology in which people are separate from environmental systems, then a Naíocene food provisioning that values life is one way to realize the web of life in agriculture.

## Writing the Naíocene into the agri-food future-present

The collection of papers in this special issue are attempts to account for issues of politics and relations amid the web of life more explicitly in contemporary agri-food studies. The first three articles build from Frank W. Geel’s socio-technical transition theory, which offers to understand transitions as non-linear processes characterized by accelerations and setbacks, surprises and unintended consequences, and political struggles and changing coalitions (Geels 2002, 2018).

In the first article, Bünger and Schiller (2022) take up a central critique of the multi-level perspective, the key heuristic of Geel’s approach, for its overall functionalist character and neglect of actors and their agency (Geels 2011; Markard et al. 2012). To overcome this critique, Bünger and Schiller develop a multi-dimensional typology of niche, regime, and hybrid actors and illustrate its usefulness for empirical research and transformative action by presenting the results of a cluster analysis based on a survey of pig and poultry farmers in Weser-Ems and Münsterland (Germany), North Brabant and Gelderland (Netherlands), and Brittany and Pays de la Loire (France). In the second article, Weituschat et al. (2022) emphasize the topic of lock-ins in their study of possible transitions toward more sustainable agri-food systems. While economic, technological, and institutional lock-ins are widely recognized in the study of socio-technical transitions (Geels 2019), the role of so-called cognitive lock-ins—mindsets and routines that hinder actors to see the benefits of alternative agricultural practices, technologies, or policies—are still under-researched. To show the importance of cognitive lock-ins, the authors focus empirically on the example of diversifying crop rotations in Cornwall (United Kingdom), and Gelderland (Netherlands) with legumes, which are well known to increase agro-biodiversity, reduce pests and diseases, and improve soil structure and fertility. In the third article, Friedrich et al. (2022) explore the role of imagination in generating more sustainable futures with respect to issues attributed to German livestock manure surpluses. Based on conceptual considerations by Adloff and Neckel (2019) they identify different trajectories of manure futures, particularly preservation, modernization, and transformation, currently shaping strategies pursued by bioeconomic innovation actors, organic farmers, non-profit organizations, and consultancies. The authors find that imagined futures (Beckert 2016) and related normative framings of the challenges discussed can be a key to overcoming effective barriers to sustainability transition.

The following two articles are concerned with the idea of shifting from the linear “take-make-consume-dispose” logic to a circular logic in agri-food systems, in which products, components, and materials stay in the value circle as long as possible by transforming into resources for other industries and reducing waste (Ellen MacArthur Foundation 2015). While the study of circular economies has been widely coined by the “economics of technological promises” (Jasanoff and Kim 2015; Giampietro 2019; Giampietro and Funtowicz 2020), the two articles approach the issue from a socio-economic perspective. In the first paper, Klein et al. (2022) examine potato producers in Lower Saxony (Germany) to shed light on the valorization of by-products as a pillar of the circular economy business model. The authors describe a shifting economic logic in the assessment of potato by-products from disposable waste to valuable resources for other sectors, which is realized through informal partnerships with livestock farmers, biogas producers, and feed companies. In the second article, Herzberg et al. (2022) emphasize the link between market power and food loss in German fruit and vegetable supply chains. While current research on food loss and waste in high-income countries predominantly focuses on consumers (Soma et al. 2021; Stenmarck et al. 2016), this article studies the under-researched early producer–retailer interface. In building on Beckert’s (2009) sociology of markets, the authors demonstrate how prevalent institutional settings privilege retailers and reinforce tendencies of food loss on part of producers that could be prevented if power asymmetries were considered more effectively in contemporary policy frameworks.

The remaining three articles of this special issue can be grouped around the notion of growth skepticism, a theoretical orientation by which scholars question the economic externalities, including care, stability, and justice, assumed by a capital growth-focused accounting of costs and benefits in world ecology (D’Alisa and Kallis 2020; Latouche 2018; Mehta and Harcourt 2021). As Gerber (2020) shows, agrarian questions consistently oppose the logics of peasant agriculture with those of Necrocene world ecology: peasant economies depend on interlinked relations of care including stable and flexible economies, secure democratic control over land and labor, and diverse ecological relations. Degrowth-oriented scholarship in the agri-food system then offers a chance to value and reorganize socioecological relationships around care and mutual aid rather than accumulation by dispossession. Michalke et al. (2022) explore how consumers within a growth-oriented economy respond to true-cost accounting, challenging the commodity veils by which labor and environment externalities are masked in German retail environments. This raises the critical political question of (1) who currently pays true prices in the agri-food system because they are exploited and (2) who ought to pay those costs because they reap the greatest rewards. In another German case study, Speck et al. (2022) show how small changes to the catering industry that incorporate agri-food externalities, here greenhouse gas emissions, could reap significant material and carbon savings. Turning attention to food producers themselves, Flachs (2022) uses degrowth metrics to explore a socioecological sustainability defined by preserving rural livelihoods and local autonomy rather than by the increased production of alternative agricultural goods. In each case, authors trouble a key element of Necrocene ontology, the apolitical externalization of economic growth from natural systems, and reckon with a more expansive vision of interconnected work in the web of life.

It should be clear that the articles collected cannot satisfyingly master the conceptual and empirical challenges that come along when approaching agri-food systems from the vantage point of the Naíocene. Nevertheless, they can be read as promising examples heading toward future directions of research and political action. As we have learned, the current crisis is a historic product of the intermingling of colonial power, profit-driven capital, and cheap nature. Built on an ontological assumption that naturalized and justified this exploitative violence, it has caused massive devastation and suffering. This historically congealed way of being in the web of life has been solidified over centuries to such an extent that capitalism is not only seen as the sole viable political and economic system worldwide, but that it is impossible even to imagine a coherent alternative to it, as Fisher (2009) put so eloquently. Against this background, the Naíocene is not comprehensively discussed in this special issue. Quite the contrary, this special issue can only be one of many needed interventions for re-assembling the agri-food systems worldwide by developing more coherent ontologies pointing at promising futures that can be translated into political action today. A radical revisioning of the true costs, politics, and daily encounters with agri-food is necessary to destabilize the dualisms of society and nature that fuel today’s multiple crises. Yet this is also an ontological opportunity, after all: all humans are part of global food ecologies, no matter how disconnected we may be made to feel through contemporary global supply chains and unequal burdens of ecological degradation. The omnipresent environmental contamination (Ahmann 2019; Guthman 2011; Udovicki et al. 2022) experienced in wealthier and poorer communities alike shows how the Necrocene comes to consume even the wellbeing of the historical beneficiaries of colonial capitalism. Care, truth, and reparation are essential steps forward in the effort to nourish and support the web of life. In other words, the Naíocene is an invitation, and all agri-food scholars worldwide are asked to participate. Join in, time is running.
